# Exploring allosteric coupling in the α-subunit of Heterotrimeric G proteins using evolutionary and ensemble-based approaches

**DOI:** 10.1186/1472-6807-8-23

**Published:** 2008-05-02

**Authors:** Kemal Sayar, Özlem Uğur, Tong Liu, Vincent J Hilser, Ongun Onaran

**Affiliations:** 1 Ankara University Faculty of Medicine, Department of Pharmacology and Clinical Pharmacology, Sıhhiye 06100, Ankara, Turkey; 2 Ankara University Faculty of Medicine, and Molecular Biology and Technology Research and Development Unit, Sıhhiye 06100, Ankara, Turkey; 3 Department of Biochemistry and Molecular Biology, and Sealy Center for Structural Biology and Molecular Biophysics, University of Texas Medical Branch, Galveston, TX, 77555-1068 USA

## Abstract

**Background:**

Allosteric coupling, which can be defined as propagation of a perturbation at one region of the protein molecule (such as ligand binding) to distant sites in the same molecule, constitutes the most general mechanism of regulation of protein function. However, unlike molecular details of ligand binding, structural elements involved in allosteric effects are difficult to diagnose. Here, we identified allosteric linkages in the α-subunits of heterotrimeric G proteins, which were evolved to transmit membrane receptor signals by allosteric mechanisms, by using two different approaches that utilize fundamentally different and independent information.

**Results:**

We analyzed: 1) correlated mutations in the family of G protein α-subunits, and 2) cooperativity of the native state ensemble of the Gαi1 or transducin. The combination of these approaches not only recovered already-known details such as the switch regions that change conformation upon nucleotide exchange, and those regions that are involved in receptor, effector or Gβγ interactions (indicating that the predictions of the analyses can be viewed with a measure of confidence), but also predicted new sites that are potentially involved in allosteric communication in the Gα protein. A summary of the new sites found in the present analysis, which were not apparent in crystallographic data, is given along with known functional and structural information. Implications of the results are discussed.

**Conclusion:**

A set of residues and/or structural elements that are potentially involved in allosteric communication in Gα is presented. This information can be used as a guide to structural, spectroscopic, mutational, and theoretical studies on the allosteric network in Gα proteins, which will provide a better understanding of G protein-mediated signal transduction.

## Background

G proteins and G protein-coupled receptors (GPCR) constitute a large family of signaling proteins that transmit extracellular signals to the intracellular milieu where the signals are integrated and transformed to a variety of biological responses. The receptor is activated by the binding of agonists, which are neurotransmitters, hormones, autacoids, odorants, taste or drug molecules present in the extracellular environment. The receptor then activates its cognate heterotrimeric G protein, which in turn transmits the signals to intracellular effectors, such as second-messenger generating enzymes or ion channels. The GDP-bound Gα subunit complexed with tightly bound Gβγ (i.e. the heterotrimer) is generally considered as the inactive state of the G protein. The agonist-activated receptor catalyses the release of bound-GDP from Gα and leads to the binding of GTP to the nucleotide binding site of Gα (nucleotide exchange). The conformational change in Gα that occurs upon nucleotide exchange results in dissociation of GTP-Gα from the Gβγ subunit. Dissociated subunits interact with downstream effectors and modulate their activity. Hydrolysis of bound-GTP to GDP by the intrinsic GTPase of Gα and subsequent association of GDP-Gα with Gβγ restore the inactive heterotrimer (see [1] for review). Accordingly, the strength of the signal transmitted to the cellular effectors is determined by the detailed kinetics and energetics of this activation-inactivation cycle. Besides the receptor, signaling proteins such as RGS (regulator of G protein signaling) [2] and AGS (activator of G protein signaling) [3] proteins are also capable of regulating or modulating G protein activation in a nontrivial manner by interfering with the GTPase activity and/or nucleotide exchange rates of Gα, or by modifying binding of the partner proteins, namely Gα, Gβγ and receptor, to each other.

**Figure 1:**
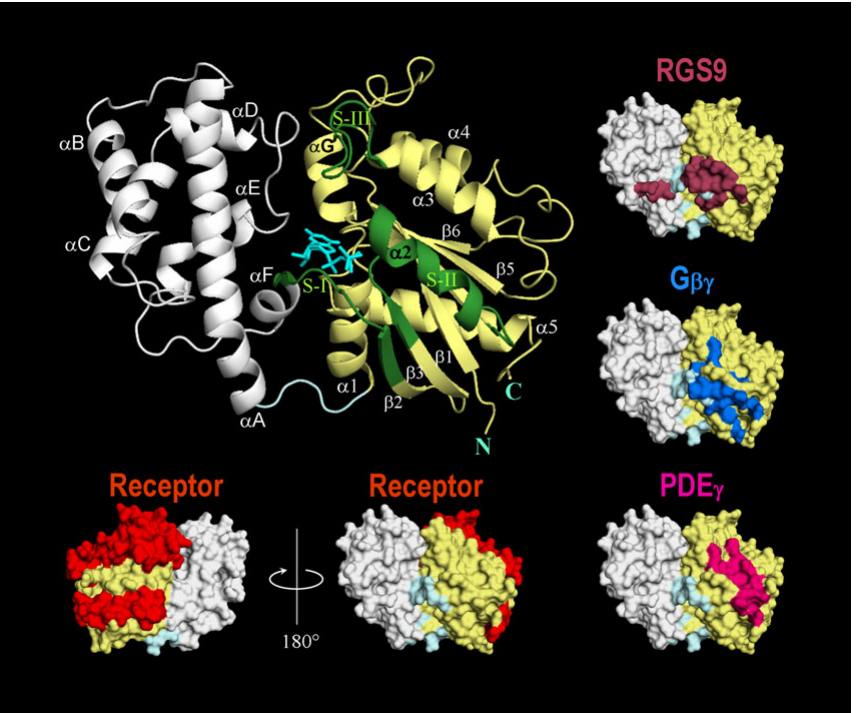
**Main structural elements of Gα subunit and its interaction sites with its partners**. Ribbon representation of transducin (Gαt) is shown in the centre as a prototypical example of Gα subunits. Ras-like GTPase and helical domains are shown as pale yellow and white, respectively. Three switch regions, indicated as S-I to S-III are colored green. Bound-nucleotide (GTPγS) is shown as cyan sticks in the centre of the molecule. The nomenclature for secondary structure is also indicated on the picture. The long N-terminal helix (26 residues) is missing in the structure. Pictures on the sides are surface representation of the molecule with the same orientation as the ribbon representation. Contact sites with Gβγ [15], RGS9 and phospodiesterase (PDEγ) [10], and sites involved in receptor recognition and interaction [1, 41, 42] are shown with different colors as indicated in the picture. All the pictures were rendered by using PyMol (DeLano Scientific LLC, South San Francisco, California, USA) with the deposited coordinates in Protein Data Bank (PDB code 1TAG-chainA).

High resolution crystal structures of different Gα subunits [4-8] have revealed that Gα consists of two main folding domains: A Ras-like GTPase domain, which is conserved in the superfamily of GTPases, with extended amino and carboxyl terminal helices, and a helical domain folded into an orthogonal bundle of six α helices which is unique to heterotrimeric G proteins. A deep guanine nucleotide binding pocket resides between the two domains that are connected with two linkers. Three flexible regions in the GTPase domain that change conformation upon nucleotide exchange have been identified and designated as switch I, II, and III (see Figure 1 for a summary). Most of the binding sites for signaling partners of Gα have been found or anticipated to be on the GTPase domain, although some of them, such as the one for RGS14 [9] or RGS9 [10], extend to the helical domain as well (see Figure 1). The interdomain interface is thought to be involved in guanine nucleotide exchange and heterotrimer dissociation (i.e. G protein activation) [11].

From a molecular point of view, regulation of G protein-mediated signal transduction relies on a complex interplay among different functional and structural domains of Gα; a perturbation at one functional domain modifies the structural, energetic and eventually functional properties of the others. Although some of these interactions are mediated by direct contacts, such as the one that takes place between RGS or AGS and nucleotide binding sites [9], most are mediated allosterically. High resolution crystal structures of Gαi [12] and Gαt [13], as well as the heterotrimers [14,15], in their GDP- or GTPγS- (a nonhydrolysable analogue of GTP) bound forms have provided remarkable insight into the understanding of guanine nucleotide-dependent conformational changes in Gα and the mechanism of GTPase. However, the realization that conformational fluctuations may play an important role in mediating allosteric coupling [16,17] casts doubt on whether a complete picture of allosteric communication can emerge from static endpoint pictures provided by crystallographic studies. Indeed, despite the wealth of available data provided by biochemical, mutational, and structural studies, many questions regarding the structural elements involved in allosteric communication remain to be answered. This is due to the fact that systematic screening of allosteric linkage, for example by using double-mutant-cycle strategies [18], is generally an intractable (if not impossible) experimental task, even for relatively small proteins.

In order to overcome these difficulties, at least in part, and to provide a structural map of a possible network of allosteric linkage in Gα, we adopted a strategy that utilizes two different computational approaches, which are based on independent and diverse assumptions and principles. We evaluated; 1) evolutionary data to draw information about statistical coupling between residues, and 2) a statistical thermodynamic model (i.e. COREX algorithm) of the native state ensemble of Gα to identify linkages in local folding free energies in Gαi and Gαt. We used currently available experimental information and the convergence of the results of the two independent analyses as a basis of judgment for the present results.

We show that the two approaches together reveal already known facts, such as the linkage between guanine nucleotide, receptor and effector binding sites, indicating that the predictions of the analyses can be viewed with a measure of confidence. Therefore, we present a set of residues and/or structural elements that may have potential functional importance for the allosteric communication in Gα, and that can be used as a guide to structural, spectroscopic, mutational, and theoretical studies on the allosteric network in Gα proteins, as well as to the interpretation of a vast amount of available experimental data.

## Methods

### Dependence of amino acid distributions in multiple sequence alignment

Correlated mutation analyses in multiple sequence alignments have long been considered as useful tools to identify sites involved in protein-protein contacts [19], or to obtain distance information for structural prediction and fold recognition [20,21]. However, Horowitz et al. [22] have observed that coordinated changes in amino acids at two different positions (i.e. compensatory mutations) in an evolutionarily sampled sequence could predict non-additivity in the corresponding double mutant cycle, which is indicative for allosteric coupling. Lockless and Ranganathan [23] later expanded on this idea and suggested that covariance information in a multiple sequence alignment (MSA) could also be used to identify long-range energetic couplings (i.e. allosteric linkage) in proteins. The reasoning was as follows: if proteins have evolved in such a way that a perturbation at one site can be allosterically transmitted to a distant site, then an evolutionary perturbation imparted by a random mutation at one of these sites should affect the evolution of the other. This should result in a dependence of amino acid distributions at the relevant sites, which could be uncovered by covariance analyses in a well sampled MSA. Although this is equivalent to saying that distant sites in a protein may have been co-evolved under common functional or structural constraints, the subtle interpretation that such associations might indicate allosteric linkage between the sites has been proved to be useful [24]. However, the free energy-like measure that has originally been suggested by Lockless and Ranganathan for this statistical coupling has potentially serious disadvantages: It is based on a virtual perturbation experiment that yields an asymmetric measure of coupling, and it is extremely biased by the conservation of the sites. The bias is due to the properties of binomial probabilities that play a central role in the measure (see supplemental information in the Additional file 1 for detailed discussion). In general, the bias is so extensive that the measure gauges conservation rather than statistical coupling. Other disadvantages have also been discussed by others [25]. In the present analysis, we therefore used a modified measure of dependence as explained below.

We assessed the dependence of amino acid distributions by means of χ^2 ^value associated with relevant contingency tables. The tables were constructed as usual. The magnitude of χ^2 ^gauges the degree of departure from independence assumption. However, the absolute magnitude of χ^2 ^depends on the actual amino acid content of the sites, which can obviously be different for every pair of sites in a protein sequence, and therefore requires normalization in order to be comparable across different pairs of sites. We used the following normalization:

$$\begin{array}{cc} \begin{array}{c} N \end{array}\chi_{ij}^{2}=2\frac{\chi_{ij}^{2}}{\left(\chi_{ii}^{2}+\chi_{jj}^{2}\right)} & \{\begin{array}{ll} i=j\Rightarrow & \begin{array}{c} N \end{array}\chi_{ij}^{2}=1\\ i\neq j\Rightarrow & 0\leq \begin{array}{c} N \end{array}\chi_{ij}^{2}<1 \end{array} \end{array}$$

The normalizing factor is the average "self-coupling" of each site, which gives the upper limit of χ^2 ^for that site. For all possible pair of sites in the MSA, $\begin{array}{c} N \end{array}\chi_{ij}^{2}$ forms a correlation-like symmetric matrix with diagonal elements equal to 1. As a further refinement we made the following correction: we calculated average $\begin{array}{c} N \end{array}\chi_{ij}^{2}$ for each pair of sites by randomly permuting the amino acids at each site in the pair (this procedure affects only the joint distribution without changing the marginals). We considered this value as the "background" $\begin{array}{c} N \end{array}\chi_{ij}^{2}$ for the sites *i *and *j*. For all possible pairs in the sequence, these values form a matrix of the same size as $\begin{array}{c} N \end{array}\chi^{2}$. We used the difference between $\begin{array}{c} N \end{array}\chi^{2}$ and the background matrix as a final measure of dependence (or statistical coupling), which can be interpreted as the extra $\begin{array}{c} N \end{array}\chi^{2}$ due to the actual arrangement of amino acids in the MSA columns. Note that χ^2 ^in this procedure was not used in its usual statistical sense, but simply as a measure of normalized "distance" between two joint distributions. Obviously, physicochemical properties of amino acids were not considered in this analysis. A comparable strategy has been undertaken by Kass and Horovitz [26]. Finally, possible contributions of evolutionary noise to the covariance information [27] were disregarded in the present analysis, since the results of COREX analysis (as explained below) were used as a source of independent information to judge the relevence of covariance data. The entire procedure is available upon request as a Matlab script.

For the present analysis, we used a non-redundant multiple sequence alignment consisting of 233 samples of α-subunits of heterotrimeric G protein sequences, obtained from the pfam database (please see Availability & requirements section below), from which redundancies were eliminated manually (the entire data set is available upon request). We excluded those sites for which the available number of amino acid samples was less than 90% of the size of the MSA. Highly conserved sites were also disregarded in the analysis since the covariance with or between such sites is statistically ambiguous as they possess little or no variance.

In order to identify the network of alike sites with respect to their coupling strength and pattern, we did a two-way hierarchical cluster analysis with the corrected $\begin{array}{c} N \end{array}\chi^{2}$ matrix after eliminating the sites that did not fulfill the sampling criterion mentioned above. We used complete linkage function and city-block distance metrics (sum of absolute differences) as the simplest and most natural choice [28]. We tested the efficiency of the entire procedure by using simulated multiple sequence alignments, in which different types of couplings with different strengths were imposed. Simulated patterns were perfectly recovered by the above procedure, whereas the original measure of Lockless and Ranganathan failed [see Additional file 1].

### Simulation of statistical ensemble

In thermodynamic equilibrium, the folded state of a protein comprises an ensemble of energetically close conformational states [29]. Each state in this ensemble emerges with a certain probability depending on its free energy. Equivalently, this ensemble can be viewed as the "state repertoire" of a single molecule that is continuously explored by the molecule with different mean dwell-times, the latter being eventually related to the state probabilities when the molecule is observed in the time scale of thermodynamic averaging. In any case, the native state of a protein for a given set of conditions can be specified by a probability distribution defined over this conformational space [30]. It can be theoretically shown that modification of this probability distribution by different perturbations made at distant sites on a protein results in energetic coupling of the perturbations when they are made simultaneously (e.g. coupling between two ligand binding processes at two distant binding sites) [31,32], which constitutes the microscopic basis of two equivalent linkage theories that have been formulated differently by Wyman [33] and Weber [34]. Thus, allosteric linkage between distant sites on a protein molecule can be analyzed by using such probability distributions [17,35], provided that the equilibrium probabilities associated with specific conformers in the native ensemble are available.

In a series of studies, Hilser, Freire and their co-workers have developed an effective strategy to model the native ensemble of folded states of proteins [30,36-38]. The procedure, known as the COREX algorithm, can be summarized as follows. The native ensemble of the protein is modeled as a collection of partially folded states of the protein: Each residue is considered as either folded (native-like as in the high-resolution structure) or unfolded (devoid of structure), and folding blocks of consecutive (usually 6 to 12) residues, in which residues are collectively folded or unfolded, are defined. The native ensemble is obtained by combinatorial folding and unfolding of these blocks. Each microstate in the ensemble thus generated is then assigned a Gibbs free energy by means of an empirically parameterized energy function [38]. This function, which is based on solvent accessible surface area and conformational entropy, has been previously calibrated and validated for globular non-membrane proteins [37]. Free energy distribution over the states (Δ*G*_*i*_) gives the probability distribution of interest:

$$\begin{array}{cc} K_{i}=e^{-\Delta G_{i}/RT} & \begin{array}{cc} Q=\sum_{i=1}^{n}K_{i} & p_{i}=\frac{K_{i}}{Q}. \end{array} \end{array}$$

*K*_*i *_is the Boltzman weight of state *i*, *Q *is the partition function of the ensemble, and *p*_*i *_is the probability of state *i*. The summation runs over the entire ensemble.

The probability distribution *p *can be used to estimate a descriptor of the residue-specific equilibrium; the residue-specific stability constant (*k*_*f*_). This quantity is the ratio of total probability of the states where residue *j *is in folded conformation to that of the states where residue *j *is in unfolded conformation:

$$k_{f,j}=\frac{\sum_{i=\text{index of states where j is folded}}p_{i}}{\sum_{i=\text{index of states where j is unfolded}}p_{i}}$$

It follows that the local folding free energy of residue *j *is -*RT*ln(*k*_*f*,*j*_). Although this quantity is designated as "local", it actually depends on all the other residues through the probability distribution, which is determined by the properties of all microstates in the ensemble to which every part of the protein contributes. Hence, in the context of a given ensemble, *k*_*f *_is local in the sense that it is a true physical descriptor of the equilibrium state of a single residue. However, this does not mean that it is independent of the state of other residues in the protein. It has been shown by extensive analysis of various proteins that these residue-specific constants reproduce a number of experimental observables, suggesting that the actual ensemble is well-represented by the COREX ensemble [38].

Another interesting piece of information that can be drawn from the COREX analysis is the degree of dependence of local folding free energies among different sites of the protein, which can be interpreted as an additional sign of allosteric linkage within the molecule [17]. This information can be obtained by evaluating folding correlations between different sites in a subset of the ensemble which contains the most probable states (e.g. with 0.99 probability):

$$r_{ij}=\frac{\mathrm{cov}\left(F_{i},F_{j}\right)}{\sqrt{\mathrm{var}\left(F_{i}\right)\mathrm{var}\left(F_{j}\right)}}.$$

*F*_*i *_and *F*_*j *_indicate the folding state (i.e. *F *= 0 or 1) of residue *i *and *j*, respectively, across the most-probable states. An alternative strategy to reveal such linkages is to calculate the propagation of thermodynamic mutations made at a specific residue to all other residues [36]. This is achieved by artificially increasing (~1 kcal/mol) the free energy of all states in which the residue of interest is folded in the representative ensemble, and then recalculating residue-specific stability constants. Such a virtual perturbation results in re-sorting of the probabilities associated with the states of the ensemble, and thus changes the value of residue-specific stability constants. Changes in the stability constants indicate how the perturbation made in a single residue propagates throughout the molecule. In principle, this information is similar to what the simple correlation analysis provides. Nevertheless, we performed both analyses, but provided only the results of the former as they indeed gave similar results.

In the present analysis we used high resolution structures of G_αi1 _and G_αt_, which have been determined in their GDP- or GTPγS-bound forms (PDB ID: 1BOF, 1GIA for G_αi1 _and 1TAG(A and B) for G_αt_). We used the web server at UTMB (please see Availability & requirements section below) for Monte-Carlo sampling of COREX states (with their calculated free energies) of G_αt_. We performed high state density (i.e., exhaustive enumeration) COREX calculations only for G_αi1_. In both cases a window size of 8 residues per folding blocks was used. In high-resolution correlation calculations we evaluated the top 5000 states of G_αi1_.

### Test statistics

In order to test whether an overlap between the two subsets of amino acid sites selected from the entire sequence was a result of pure coincidence, we used the following statistics (for the context of the problem, see results). The statistics is based on the probability distribution defined over the number of elements *x *in the intersection of two subsets selected randomly from a parent set. The null hypothesis is that the two subsets (whose sizes are given) are selected randomly. Given the size **N **of the parent set and the sizes of selected subsets **K **and **M**, the size of intersection *x *uniquely defines the partition as **N **= *n+k+m+x*, where *k *= **K**-*x, m *= **M**-*x, n *= **N-(***k*+*m*+*x*). The number of possible such partitions for a particular *x *is *q*_*x *_= **N**!/(*n*!*k*!*m*!*x*!), and for all allowed values of *x*, from 0 to min(**K**,**M**), provided that **K**+**M **≤ **N**, the total number of possible partitions is **Ω **= **Σ***q*_*x*_, {*x *= 0...min(**K**,**M**)}. Given the null hypothesis, all events in **Ω **are equally likely. Hence, the probability of selecting **K **and **M **randomly with a particular number of overlaps *x *is *p*_*x *_= *q*_*x*_/**Ω**. The statistics for the actually observed number of overlaps *x*_0 _then reads as *p*{*x *≥ *x*_0_} = **Σ***p*_*x*_, {*x *= *x*_0_...min(**K**,**M**)}.

We used *p*{*x *≥ *x*_0_} < 0.05 as a one-tail rejection criterion for the null hypothesis that the observed overlap is coincidental (or equivalently, the observed overlap is not significantly different from the expected overlap under the null hypothesis). When the alternative hypothesis is the hypothesis of interest, the left-hand tail of the same distribution can obviously be used to test whether the observed lack of overlap is coincidental or not.

## Results

In this section we briefly summarize the findings. The results of the statistical coupling and corex analyses are given separately, and then a combined picture is provided at the end. The entire matrix of statistical couplings for further analysis is available [see Additional file 2].

### Statistical couplings in G protein family

**Figure 2:**
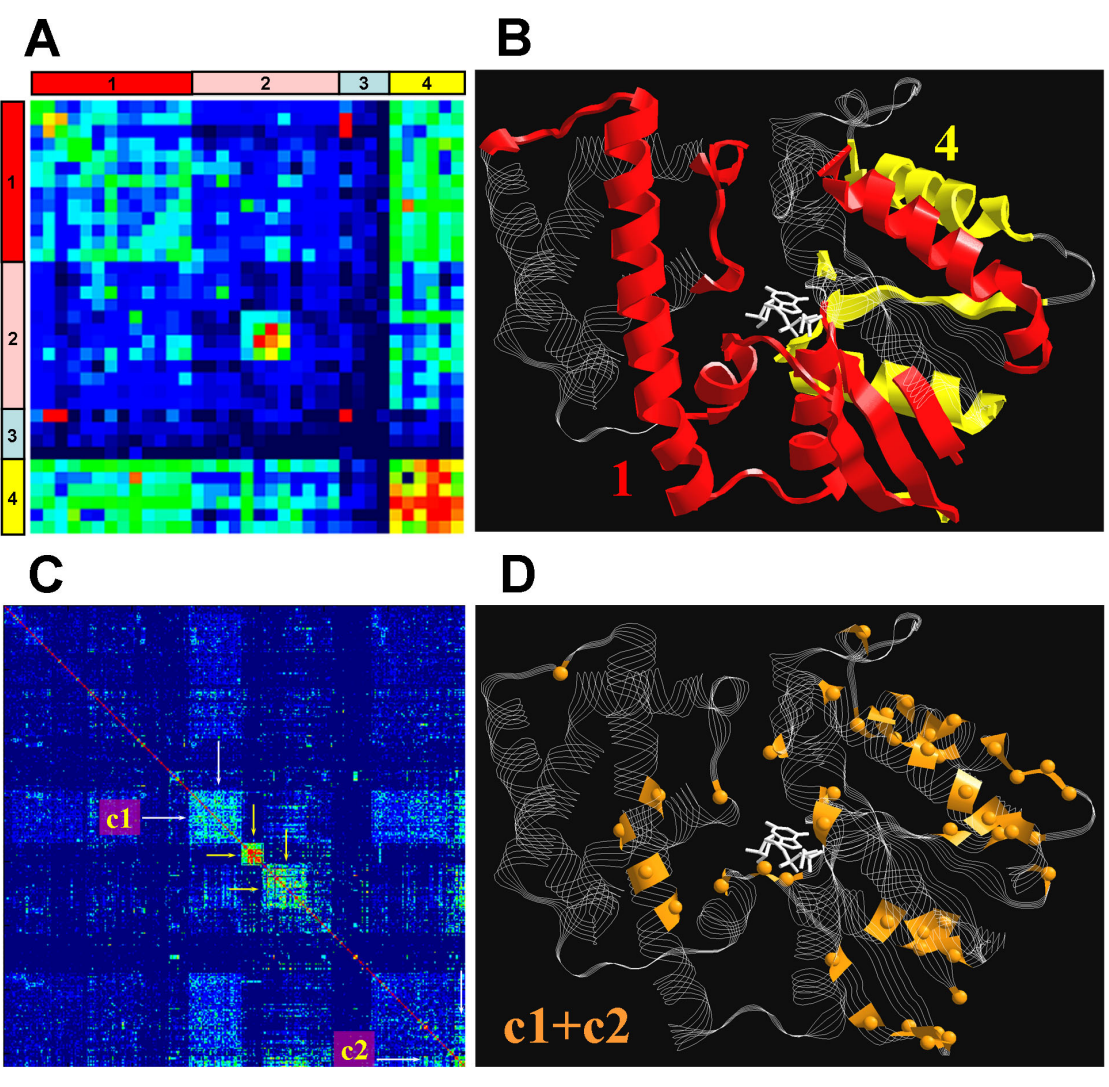
**Color coded statistical coupling matrices and mapping of selected sites on the structure of Gαi**. (A) Pair wise couplings of 35 secondary structure elements. Pixels indicate the values of statistical couplings ($\begin{array}{c} N \end{array}\chi^{2}$) on a rainbow color scale (red: full coupling, dark blue: no coupling). The secondary structure elements are sorted according to the cluster analysis and cluster numbers are indicated on the sides of the matrix. (B) The elements in clusters 1 and 4 are shown on the structure of Gαi (colors of the the numbers match the colors of the clusters in panel A). These clusters include following elements: N-terminal helix, β1, β2, β3, α1, linker I, αA, αA-αB, αD-αE, αF, linker II (switch I), α3, α3-β5 (in #1), β2-β3 hairpin tip, β5-αG, α4, β6, β6-α5, α5 (in #4). (C) The original coupling matrix (361 × 361) is sorted according to the cluster analysis. Pixels indicate the values of statistical couplings ($\begin{array}{c} N \end{array}\chi^{2}$) as in panel A. (D) Members of the two relevant clusters, indicated as c1 and c2 in panel C, are shown on the structure of Gαi as orange strips and spheres corresponding to their α-carbons. These sites correspond to (with SwissProt numbering of Gαi1): V34, V72, S75, I78, S80, F95, N149, T177, K180, V185, H188, F191, K192, L194, H195, S206, C214, A235, M240, H244, L248, S252, C254, N256, T260, K271, T284, Y296, E298, A300, A301, Q304, Q306, L310, N311, K312, R313, E318, I319, T321, T327, F334, D337, A338, T340. Coordinates of GTPγS-bound Gαi (PDB ID: 1GIA) is used to render 3D-structures. RasTop program v2.0.3 (Philippe Valadon, 2003) is used for 3D visualization.

Statistical coupling analysis in MSA provides a symmetrical matrix (361 × 361) of coupling values for all possible pairs of individual sites in G protein as described in the method section (actual number of couplings is 361 × 360/2 due to the symmetry of the matrix). However, in order to acquire a general picture of statistical coupling, we first evaluated the couplings between secondary structure blocks by averaging the coupling of individual sites in a given secondary structure element. Overall, 35 such elements as helices, sheets, loops, linkers etc. are identifiable in the molecule (13 α-helices, 6 β-sheets and 16 connecting loops and linkers). Hence, this procedure yielded a 35 × 35 symmetric matrix, entries of which represent average pairwise coupling between the secondary structure blocks (net number of couplings is 35 × 34/2). A hierarchical cluster analysis of the coupling matrix identified four clusters of elements with similar patterns and extents of couplings. The coupling matrix, rearranged according to these clusters, is shown in Figure 2A. Clusters #2 and #3 in Figure 2A consist of mainly uncoupled elements, although a mutually coupled spot is apparent in cluster #2. This spot, which is relatively isolated from the rest of the molecule in terms of statistical coupling, mainly consists of very conserved sites, whose functional importance is relatively well known (i.e. α2-helix, β4-sheet, and β4-α3 linker sequence, which correspond to switches II and III, respectively). Therefore, we did not further discuss these regions in the context of the present analysis. On the other hand, clusters #1 and #4 are formed by relatively less conserved sites. The elements in #4 are strongly, and those in #1 are moderately intra-coupled. The two clusters are also coupled to each other (see Figure 2A). Secondary structure elements that form these two clusters are shown on the structure of Gαi in Figure 2A. They cover mostly those domains that are known or predicted to be involved in the interaction of Gα with its partners, namely Gβγ, nucleotide, receptor, effector, RGS and AGS proteins (switches II and III are not present in this picture for reasons explained in the Methods section). Note that the sites in cluster 1 and 4 are located in the opposite faces of the molecule, including a part of the nucleotide binding cleft (β6-α5, β5-αG). Therefore, the observation that the elements of cluster 1 are mutually coupled to those of cluster 4 implies a statistical coupling between the two faces of the molecule. Incidentally, a mechanical coupling between the two faces of the protein has also been revealed by molecular dynamics simulations [39]. These results imply and confirm that, on average, all the functional domains of Gα are statistically (or allosterically) intercoupled. Although this may seem to be trivial at first sight, a global intercoupling of these domains implies a more complex and flexible picture of allosteric regulation than a sequential interaction scheme would suggest. For example, statistical coupling between receptor, effector and Gβγ binding sites raises the possibility of direct allosteric interactions between these sites which may by-pass the nucleotide exchange.

It is clear that secondary structure elements do not necessarily coincide with energetic or functional units in proteins [40]. Therefore, we further refined the picture given in Figure 2A by applying a cluster analysis to the coupling matrix of individual sites. The analysis identified 12 clusters of alike coupling patterns. Figure 2B shows the rearrangement of the original matrix according to these clusters. Out of twelve, two clusters designated as c1 and c2 in Figure 2C are of particular interest. These two clusters together consist of 50 moderately or poorly conserved sites (average conservation in the MSA is 40%, ranging from 19% to 86%). Moreover, these sites are slightly, albeit significantly, coupled to a considerable portion of the molecule. The sites that form cluster c1 and c2 are shown on the structure of Gαi in Figure 2B. The pattern of coupling between secondary structural elements shown in Figure 2A is well represented by the distribution of the sites that belong to c1 and c2 (compare Figure 2A and Figure 2C). Two other highly coupled clusters in the coupling matrix are marked with yellow arrows in Figure 2C. Sites in these clusters mainly correspond to the highly inter-coupled spot in cluster #2 shown in Figure 2A (i.e. mainly those sites that form switches II and III).

In summary, cluster analysis of the coupling matrix revealed a set of individual sites that were all strongly coupled to each other and moderately coupled to the rest of the molecule in the statistical sense. The overall coupling between the two domains shown in Figure 2A is well represented by these sites. Therefore, we considered these sites as the core of allosteric interactions in Gα in the statistical sense. This core, which constitutes only about 15% of the entire molecule, is designated for brevity as the "allosteric core cluster".

### Analysis of statistical thermodynamic ensemble of Gαi and Gαt

We evaluated equilibrium ensembles of Gαi and Gαt in their GDP- or GTPγS-bound forms that were modeled as partially folded states by COREX algorithm. Ensemble average of fractional folding of Gαi or Gαt showed that ~4% of the residues were unfolded in equilibrium. However, GDP-bound forms of both Gαi and Gαt were slightly less folded compared to their GTPγS-bound forms (average fractional unfolding was 5% vs. 3% in Gi, 4% vs. 3% in Gt for GDP- and GTPγS-bound forms, respectively). This was reflected by increased average conformational entropy and solvent accessible (polar and apolar) surface area in the GDP-bound form of the proteins, indicating that the GDP-bound form of Gα is slightly more "flexible" than the GTPγS-bound form in the thermodynamic sense.

**Figure 3:**
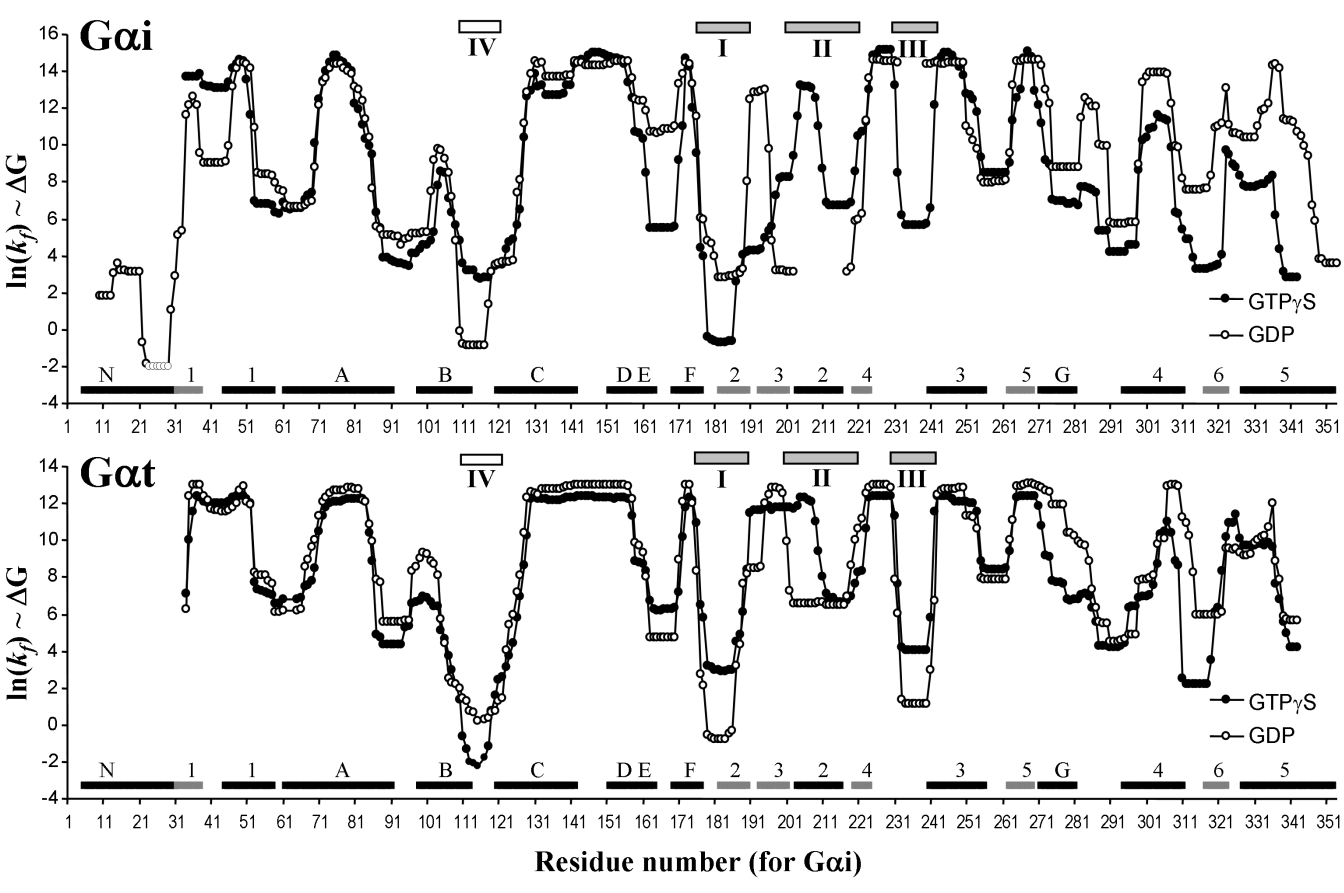
**Distribution of local folding free energies over the residues of Gαi or Gαt**. Free energies are given as ln(k_f_) in the GDP or GTPγS bound forms of the proteins as indicated. Secondary structure elements are indicated as strips next to the x-axis (black for helices, grey for β-sheets) and named according to the convention given in figure 1. Known switch regions are also shown as grey strips numbered form I to IV on top of each picture. The two sequences are aligned and Swiss Prot residue numbering for Gαi is used in both cases.

Evaluation of folding states of individual residues in the ensemble (residue-specific stability constants) showed that the local folding free energy was not distributed uniformly over the residues of Gαi or Gαt (Figure 3). The pattern of local free energy distribution was roughly similar in Gαi and Gαt, as one might expect from the structural similarity of the two proteins. In Figure 3, it is worth noting that some of the low stability regions coincide with switches I, II and III (indicated in the picture), and with the αB-αC loop, which has also been designated as a switch region in Gαi (switch-IV) that assumed nucleotide-dependent conformations.

**Figure 4:**
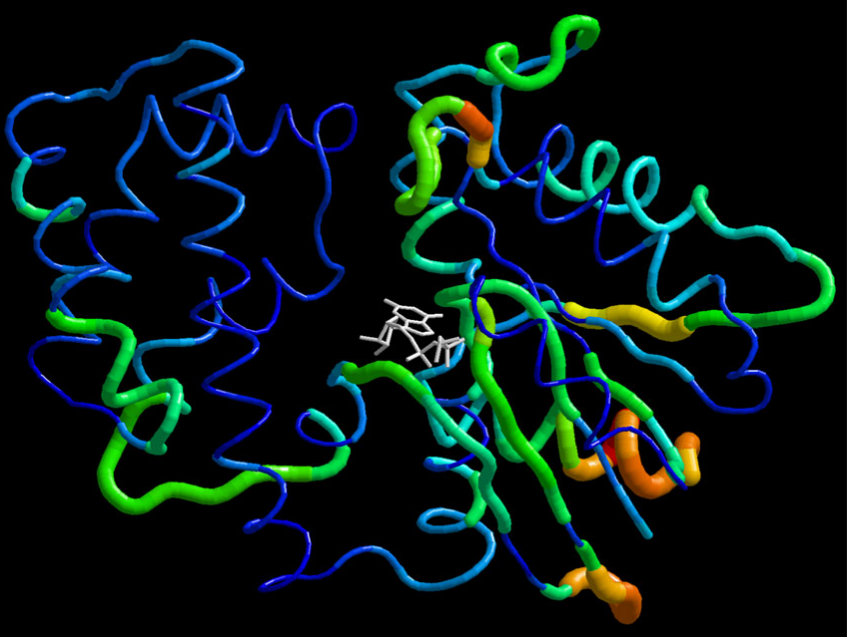
**Changes in local folding free energy upon "nucleotide exchange" on Gαi**. Changes in free energies upon nucleotide exchange in Gαi are calculated from data given in figure 3 and mapped onto the structure of Gα_i1_-GTPγS (PDB ID: 1GIA). Changes are shown on the color-coded trace-tube representation of the molecule (blue: no change – red: maximum change ~6 kcal/mol). Trace-tube radius changes proportionally to the magnitude of free energy change to help visual perception. RasTop program v2.0.3 (Philippe Valadon, 2003) is used for 3D visualization.

It is evident from Figure 3 that the local stability of many residues changes upon "nucleotide exchange", both in Gαi and Gαt. The patterns of these nucleotide-dependent changes (ΔΔG) in Gαi and Gαt are comparable. The present analysis can predict nucleotide-dependent changes in the local stability of the regions that have already been identified as switch regions in crystallographic studies (see Figure 3 for Gαt). Additional nucleotide-dependent changes that were not apparent in the crystal structure were also provided by the present analysis. The distribution of these nucleotide-dependent changes over the entire protein can be interpreted as the allosteric propagation of a free energy perturbation induced by nucleotide exchange at the guanine nucleotide binding site of Gα throughout the protein. Figure 4 shows the map of this propagation on the structure of Gαi: Perturbation upon nucleotide exchange propagates nonuniformly to distant sites in the protein, and it covers a considerable part of the molecule (especially the GTPase domain). A qualitatively similar pattern of propagation was also observable in Gαt (not shown). In addition to the switch regions, the following regions are also affected by nucleotide exchange (Figure 4): N-terminal of αG, αG-α4 loop, almost the entire sequence of α4-β6-α5, β2-β3 hairpin, and obviously a large part of the nucleotide binding site. Among these regions, αG, αG-α4 loop, α4, α4-β6 loop and α5 have all been suggested to be involved in the formation of the binding interface between Gα and receptor [1,40,41].

### Relationship between evolutionary and thermodynamic coupling

We evaluated the relationship between the residues designated by the MSA analysis as "allosteric core cluster" and the residues identified by the Corex analysis to be involved in the propagation of nucleotide-induced thermodynamic effect. We used an arbitrary cut-off of 1.5 kcal/mol (comparable with an H-bond) for the local folding free energy difference (ΔΔG) between GDP- and GTPγS-bound Gαi to construct the set of residues that were affected by nucleotide exchange. The overlap between the two sets was remarkable (ca. 65% of the allosteric core intersected with the selected set of residues for which ΔΔG>1.5 kcal/mol). However, the set of residues that changed local stability (above the cut-off) upon nucleotide exchange was quite large (about 1/3 of the entire molecule), which raised the possibility of a coincidental overlap between the two sets. We tested this possibility by using the test statistics described in the method section, which yielded a low probability of coincidence (p = 0.0086 for N = 322, K = 144, M = 45 and *x *= 28). Therefore, we interpret the observed overlap as significant, and suggest that the residues in the "allosteric core cluster" are very likely to be mediating allosteric propagation of energetic perturbations in Gα.

Additionally, we searched for folding correlations as another potential indicator of allosteric coupling in the high resolution statistical ensemble generated by the COREX algorithm for Gαi. For this analysis, 5000 partially folded states of GDP-Gαi or GTPγS-Gαi that possessed the highest probability of occurrence in the ensemble, and which accounted for a cumulative probability of greater than 99% were used. In this analysis nucleotide-dependent couplings in the molecule were disregarded, as the propagation of nucleotide-induced perturbation was already considered in the above analysis. In order to exclude the effect of nucleotide, only the correlations that are common to GDP- or GTPγS-bound forms of Gαi were selected. Also excluded were high correlations between those residues that have a high level of stability in the representative ensemble. These correlations are statistically ambiguous for reasons similar to the correlations of highly conserved (invariant) residues in the statistical coupling analysis of MSA (as mentioned above).

**Figure 5:**
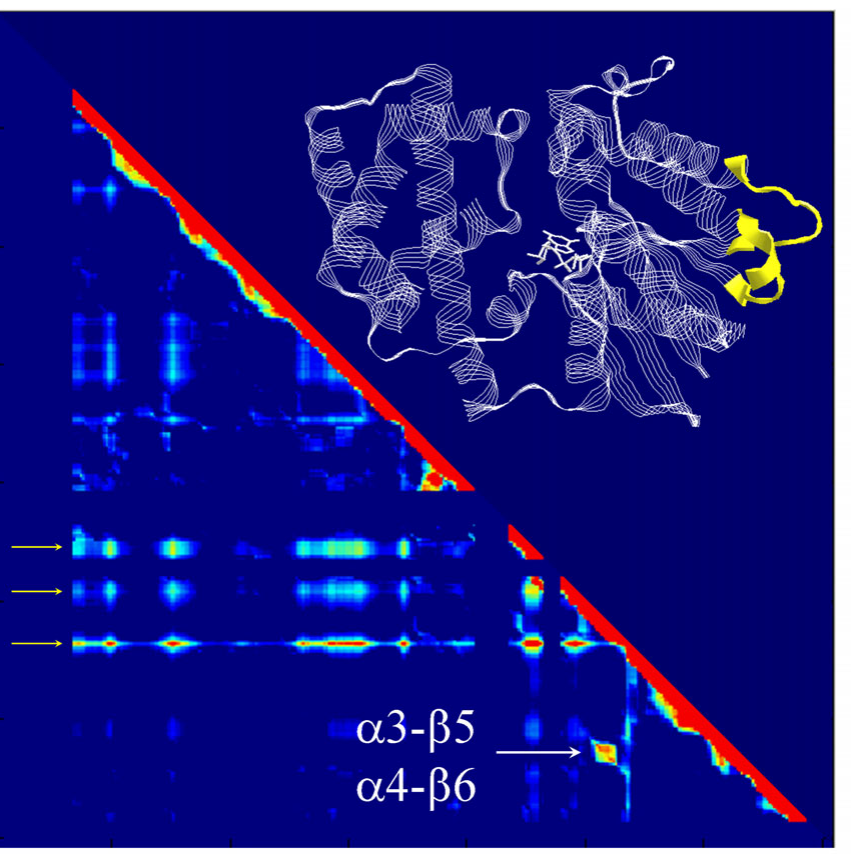
**Common folding correlations in GDP- or GTPγS-bound Gαi**. Correlation matrix is calculated from top 5000 partially folded COREX states of Gαi-GDP and Gαi-GTPγS and is shown on a rainbow color scale (blue to red correspond to 0 to 1). The matrix is constructed as explained in the text and the sites are sorted from N to C terminal (left to right, top to bottom). Only the lower triangle of the symmetric matrix is shown. Yellow arrows indicate regions of very high local stability. An unambiguous correlation group is indicated with white arrows and mapped on the structure of Giα1 (inset). RasTop program v2.0.3 (Philippe Valadon, 2003) is used for 3D visualization.

In order to filter out the correlations that were not common in GDP-Gαi and GTPγS-Gαi, we constructed a correlation matrix whose entries were obtained through element-by-element multiplication of the two correlation matrices calculated for GDP-Gαi or GTPγS-Gαi (Figure 5). In this correlation matrix, an unambiguous correlation group that corresponds to residues forming α3-β5 and α4-β6 loops was identifiable (indicated with arrows in Figure 5). The same two loops were also represented in the "allosteric core cluster" by 8 residues, and the test statistics yielded a *p *value equal to 0.0018 (for N = 322; total, K = 45; allosteric core, M = 19; correlation group, *x *= 8; overlap) indicating that the intersection between the correlation group and the "allosteric core cluster" could not be explained by pure coincidence. This again confirms the convergence of the two independent analyses, as far as the allosteric coupling is concerned. Nucleotide-independent coupling between α3-β5 and α4-β6 loops may indicate a nucleotide-independent allosteric interaction between receptor and effector on Gα, as the two loops have been implicated to be involved in effector [10] and receptor [1,41,42] interactions respectively. Such a nucleotide-independent coupling between effector and receptor sites on Gα may explain the experimental observation that β2-adrenoceptors are able to activate adenylate cyclase through Gs independently from nucleotide-exchange [43]. Using the statistical ensembles of GDP-Gαi and GTPγS-Gαi, we also performed a mutual perturbation response analysis based on thermodynamic "mutation" in the ensemble [37]. This analysis also identified the same coupling group as the one we found in the correlation analysis above (data not shown).

## Discussion

In the present study, we analyzed 1) statistical couplings in the MSA of heterotrimeric G protein family, and 2) statistical ensemble properties of Gαi and Gαt by using COREX algorithm to assess allosterically linked sites in this signal transducer family, which was not apparent in crystallographic studies. We showed that the residues captured by the two methods overlapped significantly. Considering that the information processed by the two methods was essentially different and independent, convergent results of the two analyses can be interpreted as they pointing (to a certain extent) to a common aspect of the protein sites (i.e. their involvement in allosteric communication in the protein). This is particularly important for the interpretation of statistical coupling in MSA, which is less obvious than the information provided by COREX analysis, as the covariance in MSA does not necessarily indicate allosteric coupling [44]. In this sense, the COREX algorithm seems to be a very useful tool to complement and confirm the covariance information used for this particular purpose.

**Figure 6:**
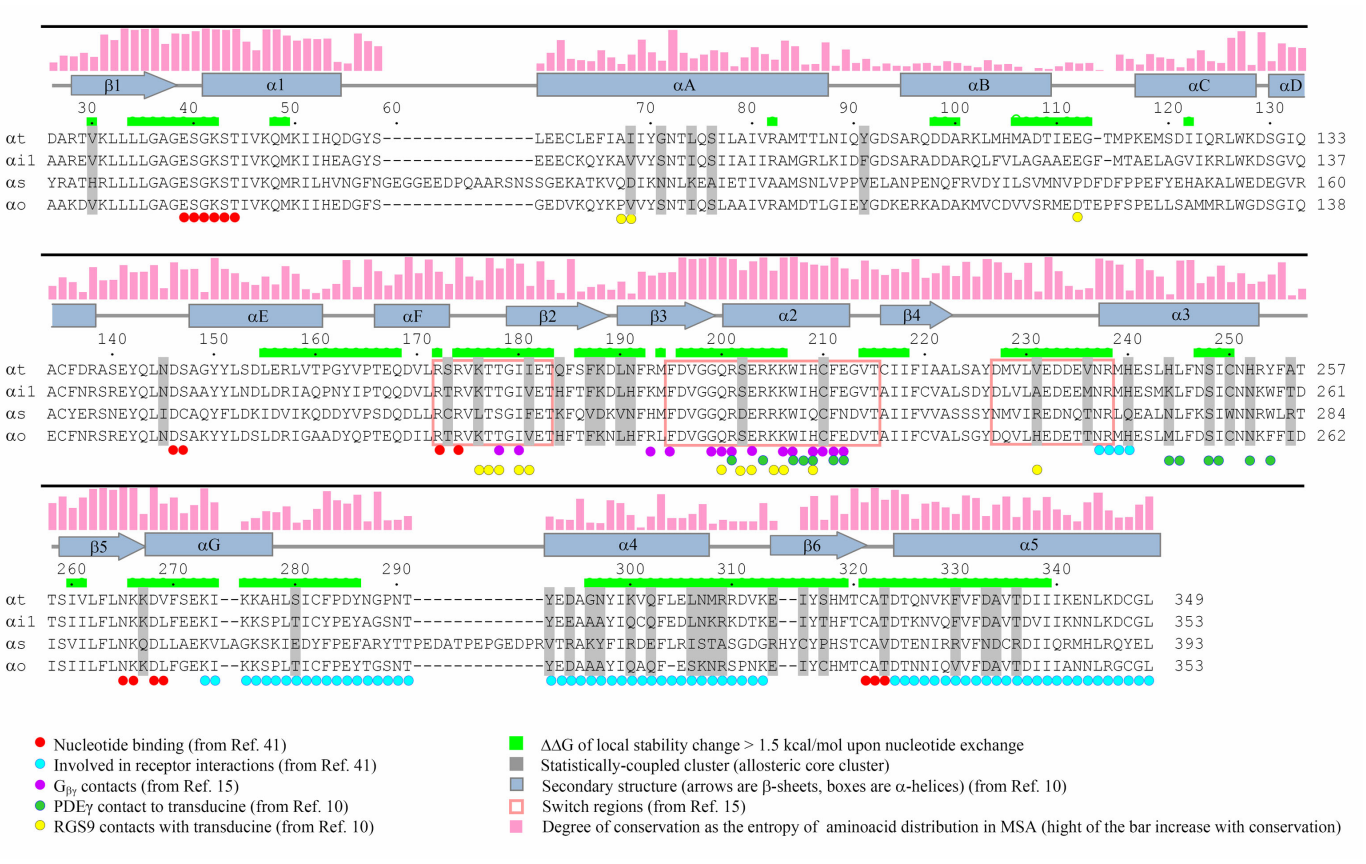
**Sites in the allosteric core cluster and residues that change stability upon nucleotide exchange**. Sites that are found in MSA and COREX analyses, along with the sites involved in known functions, are shown together on the aligned sequences of indicated Gα proteins. Coding is shown on the picture.

The results of the entire analysis, along with information available from previous analyses concerning G protein structure and function, are summarized in Figure 6 using the aligned sequences of prototypical G proteins. It is evident from Figure 6 that the residues that change stability upon nucleotide exchange (green), those that are included in the "allosteric core" (gray) and those that are located in the known functional domains of Gα (represented by different colors and symbols) show a considerable co-aggregation along the Gα sequence. Highly conserved sites (especially those that are involved in nucleotide binding and those that form switch II) do not appear in the allosteric core due to the nature of the statistical coupling analysis as discussed above. This does not mean that these sites are not involved in allosteric coupling. Conserved sites are excluded simply due to the fact that statistical coupling analysis was not appropriate to diagnose the involvement of conserved sites in allosteric linkage. Nevertheless, it is evident from COREX analysis that most sites in the switch regions (including switch-II) are thermodynamically sensitive to the identity of bound nucleotide, which implies a thermodynamic coupling between the nucleotide binding site and the switch regions (see Figures 3 and 6). Together with the observation that the folding correlation between the α3-β5 and α4-β6 loops revealed by COREX analysis was also represented significantly in the allosteric core (not indicated in Figure 6), we interpret the general picture in Figure 6 as evidence for the existence of an allosteric network that connects all (known) functional domains of Gα to each other in all possible ways (as the residues in the allosteric core are all mutually coupled). Sites in the allosteric core that have not been associated so far with an obvious function (such as those in the helical domain or in the β2-β3 hairpin tip), may then be considered as potential candidates for the mediation of intramolecular allosteric communication in the G protein family.

Two interesting observations made in the present analyses may warrant further discussion. The first one is that residues that are thermodynamically affected by nucleotide exchange and those that are represented in the allosteric core overlap much better in the GTPase domain than in the helical domain (see Figure 6). In the helical domain, COREX analysis revealed a nucleotide-induced local thermodynamic effect on the residues of switch IV and the αE-αF sequence, whereas the statistical coupling analysis hit residues along the αA helix. A trivial explanation for the observed mismatch can be that residues in the αA helix have nothing to do with allosteric linkage, as statistical coupling analysis always has the potential of such bias when it is not supported by independent information. However, it is also possible that sites identified by COREX analysis as undergoing nucleotide-dependent changes in the helical domain (i.e. switch IV and the αE-αF sequence) are not linked to other functional domains in the GTPase region as they are not covered by the allosteric core where all residues are mutually coupled. Only by experiments can these two possibilities be distinguished. The second observation is that there is a nucleotide-independent linkage between the α3-β5 and α4-β6 loops, which was revealed by both COREX and statistical coupling analyses. This suggests a direct (nucleotide-independent) coupling between effector and receptor on Gα. This linkage may result in a nucleotide exchange-independent modulation of effector-Gα interaction by the receptor. As mentioned above, experiments have shown that this may indeed be the case for β2-adrenoceptor-induced adenylyl cyclase activation through Gαs [43].

At this point, it may be worth mentioning the nature of allosteric linkage that was revealed by the present analyses. For practical purposes, the process of allosteric interactions can be seen from two different points of view: 1) The mechanistic approach, which sees the process as the propagation of a perturbation in one site to another via a series of structural distortions that extend from one site to the other, and 2) the thermodynamical or statistical mechanical approach, which considers the process as arising from the perturbation of the state distribution by ligand binding (or other effects such as mutations) in the equilibrium conformational ensemble of the protein. The latter view (to which the COREX approach belongs) can eventually represent the process only as an energetic linkage between different domains of the protein without referring to any specific mechanism whatsoever. The information provided by statistical coupling analysis in MSA also belongs to that second category. From this point of view, the effects of perturbations in one part of a protein on all other parts are due to a redistribution of the conformational ensemble [17,35]. Whether a particular substructure is stabilized by the perturbation in a particular conformational state will depend on how those ensemble-averaged properties change as a result of the redistribution. Regions (or more specifically, particular conformations of a region) that are positively coupled will be stabilized as a result of the perturbation, and regions that are negatively coupled will be destabilized [35]. In consequence, the pattern of allosteric coupling in the protein is determined by the energetic hierarchy of states (i.e. which states are the most stable and what structural and functional attributes those states possess). This may also have implications for the interpretation of mutational screening data. For example, any mutation that causes a change in the energetic hierarchy of states in the ensemble can change the allosteric coupling between two sites, even if the network of structural elements that physically connect the two sites is unaffected, which complicates the interpretation of the effect of mutation from a mechanistic point of view. Hence, the ensemble view does not imply any mechanical coupling between energetically (or statistically) linked sites. The information summarized in Figure 6 should also be understood in this context.

**Figure 7:**
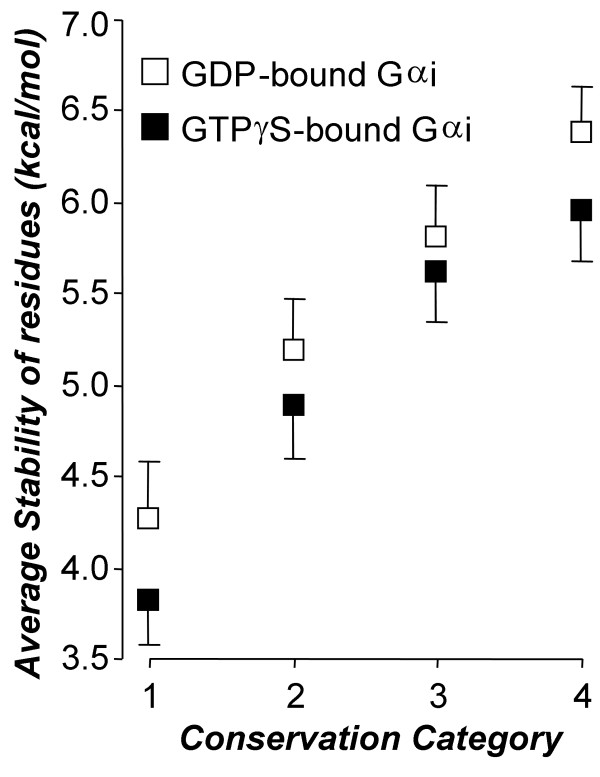
**Association between thermodynamic and evolutionary stability**. Residues are classified into four (equally-spaced) categories according to their conservation entropy in Gα-MSA (1 to 4 correspond to weakest to strongest conservation), and plotted against average residue-specific stability constants in the categories. Stability constants are calculated in GDP- or GTPγS-bound forms of Gαi. Bars are standard deviation of means. There are approximately equal numbers of sites in each category (n ≈ 80). Note that in average, there is a remarkable positive association between conservation of the residues and their local thermodynamic stabilities (local stabilities of the GDP-bound form are slightly but systematically higher than those of the GTPγS-bound form).

Another practical implication of the present analysis concerns the functional (or structural) importance that has generally been attributed to evolutionarily conserved sites in proteins. Unconserved sites have attracted less attention than the conserved for obvious reasons. However, the local conservation of the sites in the allosteric core indicated as gray-shaded residues in Figure 6 was quite weak: Average frequency of most abundant amino acids at these sites was 40%, or the average entropy of their conservations was 0.33 on a normalized scale, where 0 and 1 correspond to none and full conservation, respectively. It follows that unconserved sites may play particularly important roles in the mediation of allosteric coupling. This assertion finds a firm basis when we consider the following observation made in the present analysis: We found that conservation of sites in the G protein family, on average, scales proportionally with the thermodynamic stability of these sites within Gαi (Figure 7). In other words, there is (on average) a positive association between evolutionary and thermodynamic stability in G proteins. On the other hand, it has also been demonstrated that residues in unstable regions are important for mediating allosteric coupling [17,35]. Thus, it can be inferred that locally unconserved sites are indeed important for allosteric effects. This may explain why some allosterically important sites elude attention when we consider the fact that the literature of screening by point mutations has been mostly concentrated on conserved sites. However, this should not be understood as an implication that the conserved sites are generally unimportant in allosteric coupling.

Besides the above discussion, we believe that the observed association between evolutionary and thermodynamic stabilities is interesting in itself from a theoretical point of view, as there is no obvious reason for such an association: The former entity has to do with the variability of amino acid identity at a site in the G protein family, whereas the latter is associated with the folding stability of that site in a single member of the family (i.e. Gαi). Although a detailed discussion of the issue is beyond the scope of the present work, we may be permitted to speculate that such an association is expected (on average) in the following sense: If the protein has such a structural, energetic and functional design that a thermodynamic flexibility (rather than a particular fold) is required at a site (which is observed as a low stability of the site), then evolution may permit any amino acid at that site (which results in an unconserved site), as the folding state of that site is determined by the entire design of the protein, but not by the actual identity of the amino acid that occupies that site. The thermodynamic instability of a site may well be a part of the functional design of the protein, as discussed above. Among many other possible mechanisms, this one alone can lead to a tendency of association between evolutionary and thermodynamic stabilities of the sites. It would be interesting to investigate whether such an association exists in other protein families.

## Conclusion

We propose that the sites indicated in Figure 6 (i.e. those that are linked to nucleotide binding sites and those that are statistically coupled to each other) constitute an allosteric network in the energetic sense within the G protein. Recent progress in G protein expression, purification and spectroscopy [45-47], together with molecular biological techniques, provide a potential tool to bring the information summarized in Figure 6 to experimental test, which may eventually contribute to a more comprehensive understanding of the functional design of G proteins.

## Abbreviations

GPCR: G protein coupled receptors; RGS: regulators of G protein signaling; AGS: activator of G protein signaling; MSA: multiple sequence alignment.

## Availability & requirements

Pfam database: 

BEST @ UTMD: 

## Authors' contributions

KS performed statistical analyses and participated in the interpretation of data, TL and VJH performed high resolution COREX calculations for Giα, VJH helped in drafting the manuscript, ÖU and OO designed the study, participated in all statistical analyses, interpreted the data, and wrote the paper.

## Supplementary Material

Additional file 1 supporting information. A simulation test for the comparison of the measure of statistical coupling in MSA that was used in the present study with the one that has been originally suggested by Lockless and Ranganathan (ref 23) is provided as supporting information. Click here for file

Additional file 2 Statistical coupling matrix. Entire statistical coupling matrix for the G protein family is given as supporting data (as an MSExcel file) for further analysis. Click here for file
