# Supplementary material for: Exploring allosteric coupling in the α-subunit of Heterotrimeric G proteins using evolutionary and ensemble-based approaches
Source: BMC Struct Biol. 2008 May 2;8:23. doi: 10.1186/1472-6807-8-23 (PMC2422842; doi:10.1186/1472-6807-8-23)
Supplement: Additional file 1 — supporting information. A simulation test for the comparison of the measure of statistical coupling in MSA that was used in the present study with the one that has been originally suggested by Lockless and Ranganathan (ref 23) is provided as supporting information. [file 1472-6807-8-23-S1.pdf]

Comparison of two measures of statistical coupling

Statistical coupling analysis (SCA) suggested by Lockless and Ranganathan[1,2]

SCA is a perturbation-based method. Perturbation is achieved by selecting from full MSA a sub-alignment, which contains only the most conserved amino acid in the perturbed site. If the distribution of amino acids at another site in the sub-alignment changes significantly, then this site is said to be statistically coupled to the perturbed site. The proposed free-energy like measure of coupling of site *j* (perturbed site) to site *i* (affected site) is given as

$$\Delta\Delta G_{i,j} = kT^{*}\sqrt{\sum_{x=1}^{20}\left(\ln\frac{P^{x}_{i|\delta j}}{P^{x}_{MSA|\delta j}} - \ln\frac{P^{x}_{i}}{P^{x}_{MSA}}\right)^{2}} \quad \text{............................. eq.1}$$

where, the site indicated by *i* is represented by a 20-element vector of binomial probabilities P^x, calculated from observed counts for each amino acid (*x*=1..20), and their mean frequencies in all proteins. kT* is an arbitrary energy unit. The subscript |δ*j* indicates that the relevant probabilities are calculated after perturbing the site *j*, and the subscript *MSA* signifies a hypothetical site where 20 amino acids occur with their mean frequencies for a given number of samples. The relevant binomial probabilities are explicitly given as follows:

$$P^{x}_{i} = \binom{n}{k_x} q_x^{k_x}\left(1-q_x\right)^{(n-k_x)} \qquad P^{x}_{MSA} = \binom{n}{nq_x} q_x^{nq_x}\left(1-q_x\right)^{n(1-q_x)}$$

$$P^{x}_{i|\delta j} = \binom{n'}{k'_x} q_x^{k'_x}\left(1-q_x\right)^{(n'-k'_x)} \qquad P^{x}_{MSA|\delta j} = \binom{n'}{n'q_x} q_x^{n'q_x}\left(1-q_x\right)^{n'(1-q_x)}$$

where q^x is the mean relative frequency of amino acid of kind *x* in all proteins (given in table 2), k_x is the observed number of occurrence of the relevant amino acid at site *i*, *n* is the total number of samples at that site in MSA, and primes indicate the numbers observed in the “perturbed” subset of MSA (selected samples that contain a specific amino acid at site *j*).

The expression given in eq.1 hardly measures the statistical dependence of two sites for following reasons: Binomial probabilities used in eq.1 are sensitive to sample size and this sensitivity increases steeply when the observed amino acid frequencies deviate from their mean frequencies, which is generally the case in a given family of proteins. Note that the sample size inevitably decreases after perturbation, and the resulting effect cannot be compensated by the normalizing terms in the denominators of eq1. This behavior of binomial probabilities dominates the analysis and results in artificially high scores for conserved sites, where the deviation of amino acid frequencies from their mean values tend to be very high. Thus, $\Delta\Delta G_{i,j}^{stat}$ is expected to be highly correlated with ΔG_i^{stat} which is another energy-like measure proposed by Lockless and Ranganathan for the degree of conservation of site *i*. The artifactual nature of ΔΔG is shown in figure 1 by analyzing the same MSA for G protein-coupled receptors used by Süer et al.[2] (available from http:\\www\ghf\ghf.d).

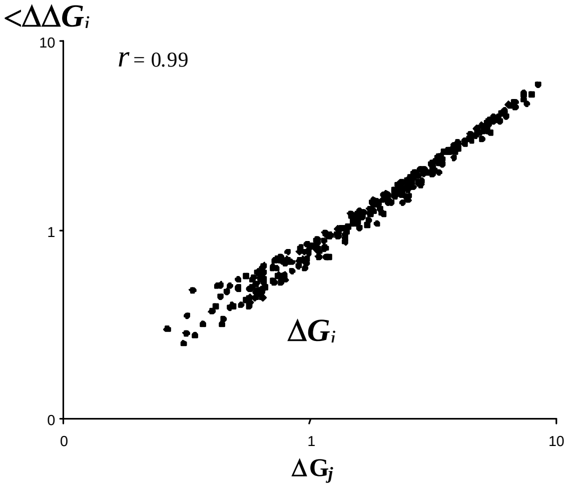

Figure 1

Relationship between mean ΔΔG values and ΔG values of the affected sites in the GPCR MSA. Mean ΔΔG for each site (represented by dots) is calculated by averaging the ΔΔG values measured by perturbing all other (allowed) sites. Data are presented on a double log scale. *r* is the estimated correlation coefficient.

In figure 1, mean ΔΔG value for each “affected” site is plotted against the ΔG value of that site. It is obvious that the expected degree of coupling of a given site to all other sites can be predicted with 99% certainty simply by looking at the

conservation status of the relevant site. Therefore, eq.1 hardly provides information about statistical coupling in general. This behavior of SCA has also been discussed by Dekker et al[3].

Dependence of amino acid distributions at two sites is actually a prerequisite for a perturbation experiment to yield a positive result. Hence, a reasonable alternative to the perturbation strategy described above is to assess directly the dependence of amino acid distributions at the pair of sites, as we did in the accompanying paper. In following lines we show that the dependence measure we propose can recover the relevant information embedded in a MSA, but the one suggested by Lockless and Ranganathan[1] fails to do so. In order to demonstrate this, we constructed a theoretical MSA consisting of 15 sites with 1000 samples of peptides. Table 1 and figure 2A show the structure of the simulated MSA. Results of the analysis are summarized in figure 2B-2E. Following are apparent in figure 2: 1) the structure given in table 1 was fully recovered when ${}_N\chi^2$ was used as a measure of coupling between sites (figure 2E). 2) The sites (1,10,11), (7,13), (8,14), (9,15) and (3,4,5,6) forms almost equally distant coupling groups while (2,12) (as they are identical) forms a separate group in cluster analysis of ${}_N\chi^2$ values, which is consistent with the simulated structure. 3) Couplings, and thus their clustering, are asymmetric in case of ΔΔG (compare the group structure in columns and rows in figure 2D). 4) ΔΔG values tend to be high as the affected sites become conserved (compare the bar graphics with the coupling matrix in figure 2B). For example, perturbation of site 1 results in high coupling with conserved sites (5,6,7,8,9,13,14,15), whereas its coupling to 10 and 11, to which it is actually coupled, is low. 5) As a result, the overall picture with ΔΔG is inconsistent with the simulated coupling structure (compare figure 2A and 2D).

Table 1 ***List of the properties of 15 sites of a simulated MSA of 1000 samples***. In the simulation, two sites were fully conserved (5, 6), six sites were moderately conserved and coupled (7, 8, 9 to 13, 14, 15 respectively), two sites were unconserved but fully coupled (2 and 12), three sites were unconserved and weakly coupled (1, 10, 11), and two sites were unconserved and uncoupled (3, 4). Unless indicated otherwise, amino acids were put randomly to each site using their mean frequency in all proteins. Amino acid frequencies used in the calculation of ΔΔG are given in **table 2**.

Site	
**1**	Random
**2**	Random
**3**	Random
**4**	Random
**5**	FULLY CONSERVED L
**6**	FULLY CONSERVED W
**7**	Random with 50% L 25% R 25% T
**8**	Random with 60% R 20% I 20% P
**9**	Random with 60% W 20% N 20% D
**10**	L if site **1** is L, random otherwise
**11**	W if site **1** is L, random otherwise
**12**	Identical with site **2**
**13**	L if site **7** is L, random otherwise
**14**	W if site **8** is R, random otherwise
**15**	W if site **9** is W, random otherwise

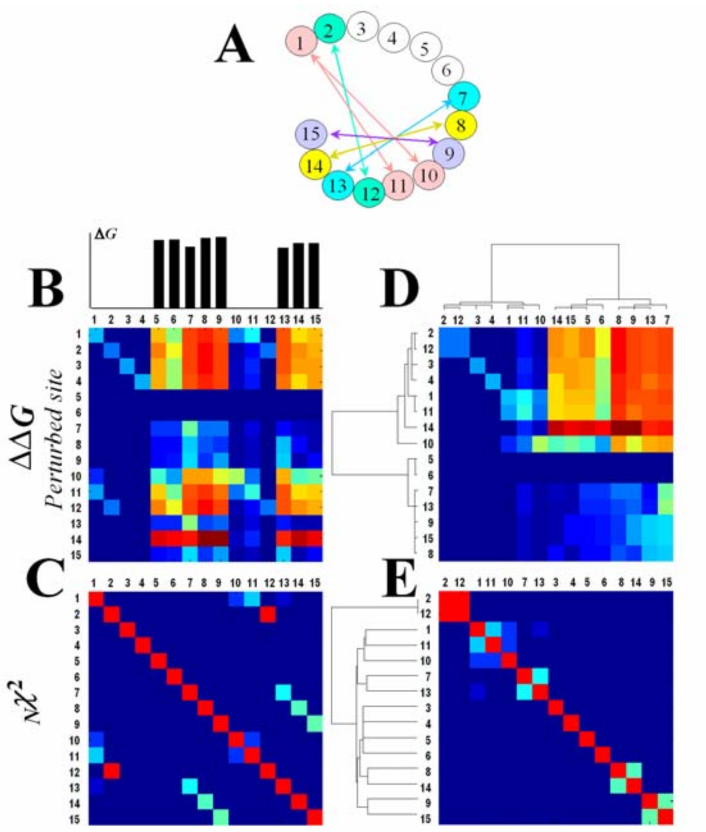

Figure 2 ***Analysis of the simulated MSA by using ΔΔG or $_N\chi^2$ as a measure of statistical coupling between sites.*** Schematic representation of the coupling structure of the simulated MSA is given in **(A)** (see also table 1). Panel **(B)** and **(C)** show the results obtained by applying ΔΔG or $_N\chi^2$ to the simulated MSA, as indicated in the picture. Data are presented as color-coded matrices of ΔΔG or $_N\chi^2$ values (blue to red for low to high values) for corresponding pair of sites. In the case of ΔΔG (**panel B**), rows and columns in the pictures represent perturbed and affected sites respectively. In panel **(B),** the conservation status of the corresponding affected-sites is also shown as a bar graphics of ΔG values on top of the coupling matrix. Panel **(D)** and **(E)** show the sorted versions of the relevant matrices (given in **B** and **C**) according to a two-way hierarchic cluster analysis (with city-block distances and complete linkages). Distance structure of the clusters is also shown next to the corresponding matrices. In the case of $_N\chi^2$ **(E)** distance-tree is only given for the rows since the matrix and its clustering are both symmetrical.

Table 2. Relative frequencies (*f*) of amino acids that were used here to calculate binomial probabilities in ΔΔG. Values are from reference 1.

Amino acid	*f*	Amino acid	*f*	Amino acid	*f*	Amino acid	*f*	Amino acid	*f*
A	0.07	F	0.04	K	0.06	P	0.05	T	0.06
C	0.03	G	0.07	L	0.09	Q	0.04	V	0.06
D	0.05	H	0.02	M	0.02	R	0.05	Y	0.01
E	0.06	I	0.05	N	0.04	S	0.07	W	0.03

In conclusion, the analysis proposed by Lockless and Ranganathan fails to extract the covariance information from MSA and any measure of statistical dependence, including the one proposed here, seems to be a better alternative to the energy-like measure that have been proposed Lockless and Ranganathan.

References

1. Lockless, S.W. & Ranganathan, R. Evolutionarily conserved pathways of energetic connectivity in protein families. *Science* **286**, 295-299 (1999).
2. Süel, G.M., Lockless, S.W., Wall, M.A. & Ranganathan, R. Evolutionarily conserved networks of residues mediate allosteric communication in proteins. *Nat. Struct. Biol.* **10**, 59-69 (2003).
3. Dekker, J.P., Fodor, A., Aldrich, R.W. & Yellen G. A perturbation-based method for calculating explicit likelyhood of evolutionary co-variance in multiple sequence allignments. *Bioinformatics* **20**, 1565-1572 (2004).
